# Supplementary material for: Differential responses of hard coral Montipora digitata and soft coral Xenia umbellata to nutrient stoichiometry under heat stress
Source: PeerJ. 2025 Nov 13;13:e20273. doi: 10.7717/peerj.20273 (PMC12619941; doi:10.7717/peerj.20273)
Supplement: Supplemental Information 3 [file peerj-13-20273-s003.docx]

Supplementary Material to

Differential responses of hard coral *Montipora digitata* and soft coral *Xenia umbellata* to nutrient stoichiometry under heat stress

Selma D. Mezger^1^, Sophie Littke^1^, Malte Ostendarp^1^, Mareike de Breuyn^1^, Christian Wild^1^

^1^ University of Bremen, Faculty of Biology and Chemistry, Marine Ecology Group, Bremen, Germany

Corresponding Author:

Selma D. Mezger^1^

Leobener Str. 6, 28359 Bremen, Germany

Email address: mezger@uni-bremen.de

**Statistical results tables for Symbiodiniaceae density data**

**Table S1.** Statistical results of testing for differences between treatments for Symbiodiniaceae counts, separated by species and experimental day.

| **Species** | **Experimental Day** | **p-value** | **Bonferroni-adjusted p** |
| --- | --- | --- | --- |
| *M. digitata* | 0 | 0.199 | 0.596 |
| *M. digitata* | 14 | 0.155 | 0.465 |
| *M. digitata* | 35 | 1.000 | 1.000 |
| *X. umbellata* | 0 | 0.309 | 0.928 |
| *X. umbellata* | 14 | 0.390 | 1.170 |
| *X. umbellata* | 35 | 0.159 | 0.476 |

**Table S2.** Statistical results of testing for differences over time within each treatment, separated by species.

| **Species** | **Treatment** | **p-value** | **Bonferroni-adjusted p** | **Significance** |
| --- | --- | --- | --- | --- |
| *M. digitata* | control | 0.0218 | 0.0653 |  |
| *M. digitata* | 5:1 | 0.0209 | **0.0209** | ***** |
| *M. digitata* | 19:1 | 0.187 | 0.561 |  |
| *X. umbellata* | control | 0.0210 | 0.0629 |  |
| *X. umbellata* | 5:1 | 0.0571 | 0.171 |  |
| *X. umbellata* | 19:1 | 0.0592 | 0.178 |  |

**Table S3.** Statistical results of testing for significant differences over time with grouped treatments, separated by species.

| **Species** | **p-value** | **Bonferroni-adjusted p** | **Significance** |
| --- | --- | --- | --- |
| *M. digitata* | 0.00309 | **0.00926** | ****** |
| *X. umbellata* | 0.000135 | **0.000405** | ******* |

**Table S4.** Pairwise posthoc comparisons for testing for significant differences over time with grouped treatments, separated by species.

| **Species** | **Comparison** | **Z** | **p-value** | **Bonferroni-adjusted p** | **Significance** |
| --- | --- | --- | --- | --- | --- |
| *M. digitata* | 0 - 14 | -0.7420 | 0.4581 | 1.0000 |  |
| *M. digitata* | 0 - 35 | 2.7262 | 0.0064 | **0.0192** | ***** |
| *M. digitata* | 14 - 35 | 3.3320 | 0.000862 | **0.002586** | ****** |
| *X. umbellata* | 0 - 14 | 3.6076 | 0.000309 | **0.000927** | ******* |
| *X. umbellata* | 0 - 35 | 3.6469 | 0.000265 | **0.000796** | ******* |
| *X. umbellata* | 14 - 35 | 0.2072 | 0.8359 | 1.0000 |  |

**Table S5.** Statistical results of testing for differences between both coral species at each experimental day with grouped treatments.

| **Experimental Day** | **p-value** | **Bonferroni-adjusted p** | **Significance** |
| --- | --- | --- | --- |
| 0 | 0.525 | 0.525 |  |
| 14 | 0.0000678 | **0.0000678** | ******* |
| 35 | 0.0170 | **0.0170** | ***** |

**Statistical results tables for Chlorophyll *a* concentration data**

**Table S6.** Statistical results of testing for differences between treatments for Chlorophyll *a* concentration, separated by species and experimental day.

| **Species** | **Experimental Day** | **p-value** | **Bonferroni-adjusted p** |
| --- | --- | --- | --- |
| *M. digitata* | 0 | 0.390 | 1.170 |
| *M. digitata* | 14 | 0.146 | 0.438 |
| *M. digitata* | 35 | 0.643 | 0.643 |
| *X. umbellata* | 0 | 0.199 | 0.596 |
| *X. umbellata* | 14 | 0.694 | 2.080 |
| *X. umbellata* | 35 | 0.175 | 0.524 |

**Table S7.** Statistical results of testing for differences over time within each treatment, separated by species.

| **Species** | **Treatment** | **p-value** | **Bonferroni-adjusted p** |
| --- | --- | --- | --- |
| *M. digitata* | control | 0.0183 | 0.0549 |
| *M. digitata* | 5:1 | 0.248 | 0.248 |
| *M. digitata* | 19:1 | 0.110 | 0.329 |
| *X. umbellata* | control | 0.735 | 2.210 |
| *X. umbellata* | 5:1 | 0.564 | 1.690 |
| *X. umbellata* | 19:1 | 0.874 | 2.620 |

**Table S8.** Statistical results of testing for significant differences over time with grouped treatments, separated by species.

| **Species** | **p-value** | **Bonferroni-adjusted p** | **Significance** |
| --- | --- | --- | --- |
| *M. digitata* | 0.000933 | **0.00280** | ****** |
| *X. umbellata* | 0.841 | 2.520 |  |

**Table S9.** Pairwise posthoc comparisons for significant differences over time with grouped treatments.

| **Species** | **Comparison** | **Z** | **p-value** | **Bonferroni-adjusted p** | **Significance** |
| --- | --- | --- | --- | --- | --- |
| *M. digitata* | 0 - 14 | -0.1391 | 0.8894 | 1.0000 |  |
| *M. digitata* | 0 - 35 | 3.3510 | 0.000805 | **0.002416** | ****** |
| *M. digitata* | 14 - 35 | 3.4646 | 0.000531 | **0.001593** | ****** |

**Table S10.** Statistical results of testing for differences between both coral species at each experimental day with grouped treatments.

| **Experimental Date** | **p-value** | **Bonferroni-adjusted p** | **Significance** |
| --- | --- | --- | --- |
| 0 | 0.773 | 0.773 |  |
| 14 | 0.0433 | **0.0433** | ***** |
| 35 | 0.00114 | **0.00114** | ****** |

**Statistical results tables for pulsation data of *X. umbellata***

**Table S11.** Statistical results of testing for differences between treatments, separated by experimental day.

| **Experimental Day** | **p-value** | **Bonferroni-adjusted p** | **Significance** |
| --- | --- | --- | --- |
| 0 | 0.901 | 2.700 |  |
| 14 | 0.0105 | **0.0315** | ***** |
| 35 | 0.0157 | **0.0471** | ***** |

**Table S12.** Pairwise posthoc comparisons for treatments at days with significant main effects.

| **Experimental Day** | **Comparison** | **Z** | **p-value** | **Bonferroni-adjusted p** | **Significance** |
| --- | --- | --- | --- | --- | --- |
| 14 | 19:1 - 5:1 | 0.8374 | 0.4024 | 1.0000 |  |
| 14 | 19:1 - control | -2.0934 | 0.0363 | 0.1089 |  |
| 14 | 5:1 - control | -2.9308 | 0.0034 | **0.0101** | ***** |
| 35 | 19:1 - 5:1 | 0.0000 | 1.0000 | 1.0000 |  |
| 35 | 19:1 - control | -2.6312 | 0.0085 | **0.0255** | ***** |
| 35 | 5:1 - control | -2.1483 | 0.0317 | 0.0951 |  |

**Table S13.** Statistical results of testing for differences over time within each treatment.

| **Treatment** | **p-value** | **Bonferroni-adjusted p** | **Significance** |
| --- | --- | --- | --- |
| control | 0.0277 | 0.0830 |  |
| 5:1 | 0.0157 | **0.0471** | ***** |
| 19:1 | 0.0125 | **0.0376** | ***** |

**Table S14.** Pairwise posthoc comparisons for days within treatments with significant main effects.

| **Treatment** | **Comparison** | **Z** | **p-value** | **Bonferroni-adjusted p** | **Significance** |
| --- | --- | --- | --- | --- | --- |
| 5:1 | 0 - 14 | 2.6312 | 0.0085 | **0.0255** | ***** |
| 5:1 | 0 - 35 | 2.1483 | 0.0317 | 0.0951 |  |
| 5:1 | 14 - 35 | 0.0000 | 1.0000 | 1.0000 |  |
| 19:1 | 0 - 14 | 1.9967 | 0.0459 | 0.1376 |  |
| 19:1 | 0 - 35 | 2.8900 | 0.0039 | **0.0116** | ***** |
| 19:1 | 14 - 35 | 0.8933 | 0.3717 | 1.0000 |  |

**Statistical results table for survival data**

**Table S15.** Pairwise posthoc comparisons for survival between treatments at the end of the experiment.

| **Species** | **Comparison** | **BH-adjusted p-value** | **Significance** |
| --- | --- | --- | --- |
| *M. digitata* | control - 5:1 | **3.9 × 10⁻⁸** | ******* |
| *M. digitata* | control - 19:1 | **7.2 × 10⁻⁷** | ******* |
| *M. digitata* | 5:1 - 19:1 | 0.095 |  |
| *X. umbellata* | control - 5:1 | **3.8 × 10⁻⁹** | ******* |
| *X. umbellata* | control - 19:1 | **0.00256** | ****** |
| *X. umbellata* | 5:1 - 19:1 | **0.00037** | ******* |
