# Supplementary material for: Differential responses of hard coral Montipora digitata and soft coral Xenia umbellata to nutrient stoichiometry under heat stress
Source: PeerJ. 2025 Nov 13;13:e20273. doi: 10.7717/peerj.20273 (PMC12619941; doi:10.7717/peerj.20273)
Supplement: Supplemental Information 4 [file peerj-13-20273-s004.docx]

**Codebook**

**to the Supplemetary File “Supplementary_Table_RAWdata”**

Sheet “Survival”:

Use as factor:

- Tank (tank number the sample was from)
- Species (Coral species)
- Treatment (LN/LP = control; HN/HP = 5:1 ratio; HN/MP = 19:1 ratio)
- SampleID

Use as numeric value:

- Survival_Time
- Status (regarding Kaplan-Meier curves: 0 = coral was still alive (or censored) at that given Survival_Time, 1 = coral died at that given Survival_Time)

Sheet “Pulsation”

Use as factor:

- Experimental_Date (Experimental day)
- Date (specific date of the day)
- Tank (tank number the sample was from)
- Treatment (control = control; 8PO4 = 5:1 ratio; 2PO4 = 19:1 ratio)

Use as numeric value:

- Polyp1 (pulsation of 1st polyp over the time of 30 sec)
- Polyp 2 (pulsation of 2nd polyp over the time of 30 sec)
- Polyp 3 (pulsation of 3rd polyp over the time of 30 sec)
- Mean_Puls (calculated mean pulsation rate of polyp1-3 over the time of 30 sec)

Sheet “Symbiodiniaceae”

Use as factor:

- Day (Experimental day)
- Treatment (control = control; 8PO4 = 5:1 ratio; 2PO4 = 19:1 ratio)
- Tank (tank number the sample was from)
- Species (Coral species)

Use as numeric value:

- Mean_Zoox_Density (Mean Symbiodinaceae density normalized to surface area

Sheet “Chl a”

Use as factor:

- Experimental_Date (Experimental day)
- Treatment (control = control; 8PO4 = 5:1 ratio; 2PO4 = 19:1 ratio)
- Tank (tank number the sample was from)
- Species (Coral species)

Use as numeric value:

- Surface area cm2 (fragment surface area in cm^2^)
- Sample Volume (mL) (Sample tissue slurry volume – was used to standardize Chl a content and Symbiodiniaceae density in earlier steps)
- Mean_Chla_(pg/algal cell) (Mean chlorophyll a concentration per Symbiodiniaceae)
- Mean_Zoox_Density (Mean Symbiodinaceae density normalized to surface area
- Mean_Chla_(pg/cm) (Mean chlorophyll a concentration per surface area – was used for some explorative work but not used in the manuscript)
